# Colorectal cancer metastasis: in the surgeon's hands?

**DOI:** 10.1186/1477-7800-2-5

**Published:** 2005-02-24

**Authors:** Gary Atkin, Abhay Chopada, Ian Mitchell

**Affiliations:** 1 Department of Surgery, Barnet General Hospital, Wellhouse Lane, Herts, EN5 3DJ, UK; 2 Department of Surgery, Ealing Hospital, Uxbridge Rd, Middlesex, UB1 3HW, UK

## Abstract

**Background:**

Lymphovascular ligation before tumour manipulation during colorectal cancer resection is termed the '*no-touch isolation*' technique. It aims to reduce the intra-operative dissemination of colorectal cancer cells. Recently, the detection of circulating tumour cells has been enhanced by molecular biology techniques. This paper reviews the evidence for the no-touch isolation technique in light of the recent developments in circulating tumour cell detection.

**Methods:**

Studies investigating the effect of colorectal cancer surgery on circulating tumour cells were identified by a Medline search using the subject headings colorectal neoplasms and neoplasm circulating cells together with the map term 'no-touch isolation technique'. Further references were obtained from key articles.

**Results:**

Molecular biological techniques have improved the detection of circulating colorectal cancer cells. There is a trend towards reduced tumour cell dissemination with the no-touch technique compared with the conventional method. However the benefit in terms of improved patient survival remains unproven.

**Conclusion:**

The no-touch isolation technique reduces circulating tumour cell dissemination but further work is needed to determine the significance of this with regards to patient survival.

## Background

Of the patients with colorectal cancer (CRC) undergoing surgery for resectable disease, 30–50% will subsequently develop metastases [1]. Dissemination of tumour cells is therefore thought to occur early on in the disease process. The principle of early lymphovascular ligation before manipulation of the tumour during the surgical resection of a CRC has been termed the '*no-touch isolation*' technique. This was proposed by Barnes [2] as a way of reducing the incidence of liver metastases by diminishing the intra-operative dissemination of CRC cells. An early proponent of the technique was Turnbull et al [3], but their findings have not been confirmed in other studies [4].

Animal studies suggest tumour cells are shed into the circulation during resection of a primary tumour, increasing the likelihood of metastases [5]. However, the evidence in humans is not clear. This may be because the early techniques used to detect circulating tumour cells (CTC) were not sufficiently sensitive to detect the small number of cancer cells present within the blood. These detection methods relied on cytological examination of blood smears, and allowed the detection of 1 tumour cell within 100 normal cells [6].

In recent years, the question of tumour cell dissemination during surgical resection has been re-examined following the introduction of molecular biology techniques to detect CTC. These are much more sensitive, and it is now possible to detect one tumour cell in a sample of 10^7 ^normal cells [7].

This paper reviews the evidence for the no-touch isolation technique for the resection of a CRC in light of the recent developments in molecular biological techniques for CTC detection.

## Methods

In performing this review, the studies looking at the effect of CRC surgery on CTC were identified by a Medline search using the subject headings colorectal neoplasms and neoplasm circulating cells together with the map term 'no-touch isolation technique' [8-16]. Further references were obtained from key articles [17-20].

We decided to review this topic by addressing three areas of uncertainty. Firstly, does the surgical manipulation of a CRC increase the level of tumour cells within the blood? Secondly, what is the biological significance of CTC in CRC? Thirdly, is the no-touch isolation technique of CRC resection associated with an improved patient survival?

To investigate the effect of tumour manipulation on CTC, we have reported the conversion, for each reviewed article, of a negative preoperative blood sample (for CTC) to a positive intra- or post-operative sample within the same patient. We have only noted conversions within the same sample source (systemic venous (SV) or portal venous (PV) blood). Where conversion rates cannot be determined, as data for individual patients are not provided, a comparison between overall preoperative and intra/post-operative positivity for CTC is given. Unless otherwise stated in the reviewed article, we have presumed the conventional method was utilised (tumour manipulation before lymphovascular ligation).

## Results

### A) Does the surgical manipulation of a CRC increase the level of tumour cells within the blood?

A number of experimental models have been used to investigate the effect of surgical manipulation on CTC. Nishitaki et al [5] studied 2 groups of rabbits with surgically-inoculated liver tumours. The first group had a laparotomy and manipulation of the hepatic tumour 14 days after inoculation, together with resection of the tumour. The second group had a laparotomy and resection of the tumour but without manipulation. Two weeks later the rabbits were sacrificed, and pathological examination showed significantly more hepatic venous invasion by cancer cells in the tumour manipulation group, together with significantly more lung metastases and shorter mean survival.

Romsdahl et al [21] measured the number of tumour cells by cytological examination in blood samples from the inferior vena cava of rats during manipulation of a thigh tumour. There was a manifold increase in the number of CTC during manipulation, which decreased rapidly, so that 4 minutes after manipulation 93–96% of the cells had disappeared from the circulation. In a similar experiment with mice, Liotta et al [22] showed a 10-fold increase in tumour cells in the venous effluent of a thigh tumour during manipulation.

In order for the no-touch technique to be effective in humans, early ligation of the main pedicle must eliminate passage of liberated tumour cells into the portal and systemic circulations. However, Salsbury et al [23] found the number of tumour cells in the common iliac vein dramatically increased after ligation of the inferior mesenteric vein, suggesting there was a shift of the draining venous blood into the systemic circulation following ligation. In an experimental model using dogs, Ackerman [24] showed clamping of the mesenteric veins produced an increased intestinal lymphatic and venous outflow, thereby enhancing the possibility of tumour cell dissemination. He concluded ligation of the major arterial supply, followed by the marginal vessels, is the most effective technique in minimising outflow.

On theoretical grounds, the no-touch technique would appear unsuitable for low rectal cancers, as it is necessary to mobilise tumours in the rectum before all the draining veins are controlled. In addition, a large proportion of the venous drainage occurs into the iliac system, thereby negating the effect of mesenteric pedicle ligation.

#### Results of reviewed articles

There have been numerous studies investigating the effect of tumour manipulation on CTC in human tumours. This review focuses on studies involving CRC. The overall conversion rates for each study (ie conversion from negative preoperatively for CTC to positive intra or post-operatively taking into account all study patients) are listed in Additional Files 1 and 2. The conversion rates taking in to account only those patients negative preoperatively for CTC are also listed.

The only study to compare directly the conventional and no-touch techniques for the resection of a CRC involved the non-random assignment of 27 patients into conventional and no-touch groups [8]. The technique of mutant-allele-specific amplification (MASA) was used to identify mutations of K-ras or p53 genes in the primary tumour of each patient. Cells containing the same mutations were then examined for in blood samples taken before, during, and after surgery. In the conventional group, 11 patients had K-ras or p53 mutations in their primary tumour. None of these patients had preoperative blood samples that contained cells exhibiting similar mutations. However, 8 (73%) did have cells containing similar mutations in the blood taken intra-operatively following tumour manipulation. In the no-touch group, 7 patients had mutations in their primary tumour. None of these patients showed similar mutations preoperatively, and only 1 patient (14%) became positive intra-operatively. From these findings, the authors conclude the no-touch technique is effective at reducing the intra-operative dissemination of colorectal tumour cells.

The other studies involving CRC have used either the conventional or no-touch surgical techniques, but have not directly compared both. Weitz et al [9] and Sales et al [10] employed the no-touch technique and found an overall conversion rate of 16% and 9% respectively. Both studies conclude the no-touch technique is effective at reducing tumour cell dissemination. Bessa et al [17] also used the no-touch technique in 50 patients who were randomly assigned to undergo open or laparoscopically-assisted colectomy. They found an overall conversion rate of 8% and 12% respectively in SV samples.

Studies employing the conventional technique of CRC resection have found overall conversion rates of 0 – 80% in SV samples [11-15,18,19] (see Additional file 2). Funaki et al [20] demonstrated the highest conversion, with 4 out of a study sample of 5 patients converting intra-operatively. This is much higher than in any other study, and although the sample size is small, the authors suggest this is evidence of tumour cell dissemination as a result of surgical manipulation. It is interesting to note 80% of these patients had rectal cancers, which require significant mobilisation before control of the venous output is obtained. Griffiths et al [18] did not detail individual patients and so the conversion rate cannot be determined, but they found 4% of patients were positive preoperatively, 50% were positive intra-operatively, and 8% postoperatively. Similarly, Ito et al [15] found significantly more patients were positive for CTC following surgery compared with preoperatively (51% v 38%, p = 0.003). Most authors conclude their results demonstrate evidence of tumour cell dissemination. However, Garcia-Olmo et al [11], who used the conventional technique and did not demonstrate a conversion in any patients, concluded the no-touch isolation technique was unnecessary.

As a number of patients were positive for CTC before surgery, a more direct assessment of the effect of tumour manipulation might be to analyse the conversion rates only for those patients negative for CTC preoperatively. This reveals a much greater conversion rate for most studies (see Additional Files 1 and 2). For example, using the conventional method, Funaki et al [20] found all 4 patients negative preoperatively converted intra-operatively. Similarly, Tien et al [13] demonstrated a conversion of 49% for SV, and 52% for PV samples. Conversely, Garcia-Olmo et al [11] used the conventional method and failed to show conversion in any patients. For the no-touch technique, the conversion rates are also greater if only the patients negative preoperatively are considered. Bessa et al [17] demonstrated the highest conversion rate, with 50% and 33% of patients converting following laparoscopically-assisted colectomy within SV and PV samples respectively.

#### Limitations and variations of studies under review

##### 1) Choice of technique used to detect CTC

Before the advent of molecular biology, the detection of tumour cells within the blood depended on cytological examination of a blood smear. The incidence of circulating tumour cells during CRC resection detected by cytological examination of blood smears has been reported as 25–67% [25,26]. Griffiths et al [18] state it is easy to detect CTC within a blood sample, but this method of detection underestimates the true number of circulating cells as there is probably a considerable loss in the preparation of blood cell concentrates. Also, difficulties in interpretation arise when other large mononuclear cells mimicking cancer cells are present.

Immunocytochemistry has been used to detect CTC in a number of tumour types, including colorectal cancer [27]. This technique labels tumour cells with antibodies against specific cell components that are not expressed in haematopoietic cells. It has the advantages of preserving cellular morphology and allowing identification of cell clusters, an important drawback of molecular detection techniques [28]. It also permits simultaneous assessment of other cellular details such as proliferation activity and oncogene expression. However, it is labour intensive due to the low concentration of CTC. The sensitivity has also been questioned [29], although this can be enhanced with flow cytometry.

In recent years, molecular biological techniques have been developed for use in tumour cell detection. The polymerase chain reaction (PCR) involves detection and amplification of certain DNA mutations. A copy of the DNA containing the target sequence is made and the process is repeated many times, producing multiple copies and thereby increasing the sensitivity of detection. Mutations in the k-ras gene have often been used for detection of CTC in CRC.

However, the detection of these sequences within the blood does not necessarily imply there are circulating tumour cells, as DNA is relatively stable and may represent fragments of tumour DNA released by cell necrosis or apoptosis and persisting in the blood for some time [30]. Also, this method of detection relies on the presence of specific DNA mutations within the primary tumour, and the genetic changes associated with CRC are known to be heterogenous [28].

An alternative technique, now more commonly employed, is reverse transcriptase-PCR (RT-PCR). This detects messenger RNA (mRNA), which is used as an indicator of tissue-specific gene transcription. The technique is rapid, more easily automated than immunocytochemistry, and is capable of detecting one tumour cell in 10^7 ^normal cells [19]. RNA is rapidly degraded and so its presence in the blood suggests active expression by circulating tumour cells [13].

However, there is a lack of standardisation of RT-PCR techniques, resulting in different sensitivities and specificities between centres [31]. Illegitimate transcription (when tissue-specific genes are transcribed in non-specific cells) can occur, and the presence of non-malignant epithelial cells in the blood (for example, following venepuncture [32]) reduces the specificity. RT-PCR detects the number of copies of mRNA, not the number of CTC, so it may not accurately assess an increase in tumour cells secondary to mobilisation [13]; also it may be difficult to derive the prognostic value of CTC if the levels cannot be accurately determined in individual patients. False negatives can occur with RT-PCR, affecting the technique's sensitivity. The marker of interest may not be expressed because of tumour cell heterogeneity or because there is a poorly-differentiated subclone that has lost the ability to express the tissue-specific marker [33]. PCR inhibitors may be present within some body tissues, and it is important to note the *in-vitro *sensitivity reported in the reviewed articles will be higher than the true *in-vivo *sensitivity, as the cell lines used to determine the sensitivity are known to express strongly the marker of interest and are not affected by the presence of *in-vivo *PCR inhibitors.

Most of the reviewed articles that state the specificity of their technique claim a rate of 100%, indicating all the tumour samples were positive for the marker of interest, whereas none of the control blood samples (from healthy volunteers or patients undergoing resection for benign disease) were positive. However, Patel et al [14] examined 143 control subjects and found 7 (5%) were positive when a single blood sample was assessed for CTC.

##### 2) Choice of marker used to detect CTC

There are no tumour-specific genes for CRC, and it is known malignant cells continue to express markers that are characteristic of their tissue of origin. Therefore, a range of epithelial cell markers, which are normally absent from the blood, have been used as RT-PCR targets for cancer cells derived from epithelial tissues.

The cytokeratin (CK) polypeptides are found within the cytoplasm of epithelial cells, and several CK markers have been used to detect CTC. The most common of these is CK 20, which is expressed in gastrointestinal epithelia, urothelium, Merkel cells, and tumours derived from these tissues [9]. Carcinoembryonic antigen (CEA) is a glycoprotein found on the surface of epithelial cells. It is overexpressed in nearly all colorectal cancers [34], as well as other tumours such as breast and non-small cell lung carcinoma, and has been widely used to detect CTC. Guanylyl cyclase C (GCC) is another tissue-specific marker only expressed in normal intestinal mucosa. It is a member of the guanylyl cyclase family of receptors, and its expression persists once the epithelial cell has undergone neoplastic transformation [13]. Cell surface sialylated carbohydrates, such as sialyl Le^a ^and sialyl Le^x^, are associated with CRC formation and progression [35], and have been used to detect disseminated tumour cells as well as predict prognosis.

Concerns regarding marker specificity have been raised. CK 8, 18 and 19 have all been shown to be expressed in normal blood [29]. CK 20 and CEA are more specific, although they have also been seen in control samples [31,36]. The CEA family includes homologous genes expressed in granulocytes [37], and CEA may be induced by surgical stress [15] as well as occurring in the blood of patients with inflammatory conditions such as colitis [38]. GCC appears to be the most specific marker investigated. Bustin et al [31] found CK 20 transcripts in all 21 healthy volunteers studied, whereas GCC was found in only a single control.

##### 3) Choice of blood sample used to detect CTC

The source of blood for analysis appears to be important. Most studies have investigated systemic venous samples for the presence of CTC. Animal studies suggest tumour cells become trapped in the capillary bed of the first organ encountered [39,40], and so for CRC would be seen in the liver but not in peripheral blood. Theoretically, CRC cells must pass through the liver, lungs and the microcirculation of the other tissues of the body before they pass into the systemic venous circulation. Also, any CTC should be diluted by the larger blood volume of the peripheral circulation [12]. There should be many more circulating colorectal tumour cells in the portal circulation than the systemic circulation, therefore. Griffiths et al [18] found 57% of patients were positive for CTC in PV blood during resection a colorectal tumour, as opposed to 50% of patients who were positive in their SV samples. Tien et al [13] found more PV samples were positive before, during and after surgery compared with SV samples. However, 3 of the 17 who were positive in the peripheral blood were negative in all portal samples, suggesting tumour cells were bypassing the portal circulation and entering the systemic circulation directly. It is interesting to note these 3 patients had Dukes stage C disease and it may be that tumour cells were passing directly from the lymphatics into the peripheral circulation. Bessa et al [17] found there was concordance between SV and PV samples for CEA mRNA in only 65% of cases. They also found no patients demonstrated a conversion in PV samples following open colectomy, however 8% converted in SV blood.

Due to the discrepancy in sample sources, we have only noted patients who converted within the same venous compartment. Several studies detected CTC in draining vein blood intra-operatively, and compared it against a reference SV sample taken before manipulation. It is difficult to be certain of conversion in these patients, however, as it is possible the PV samples were positive preoperatively. It is important for subsequent studies to measure CTC in both systemic venous and draining vein blood, before, during and after tumour manipulation to investigate further the discrepancy in CTC detection between the venous compartments.

The importance of multiple sampling should be considered. Glaves et al [41] suggests cancer cells are intermittently shed in to the blood, so sampling errors may occur if single samples are taken. Tien et al [13] sampled SV and PV blood twice during tumour mobilisation and found that of the 14 patients who were positive in portal blood during mobilisation, 5 (36%) were only positive in the second sample, and so would have been considered negative if a single sample was taken. In the SV samples, 3 out of 17 (18%) patients were only positive in the second sample. Mori et al [19] also performed sequential sampling intra-operatively, and found of the 5 patients positive for CTC at any of the 4 sampling time points, 2 (40%) were positive at a single time point only. Wharton et al [42] showed by increasing the sampling frequency (from once to twice), the detection of CTC is significantly increased.

Weitz et al [9] suggest another factor affecting the detection of CTC may be the degree of intra-operative blood loss and subsequent administration of intravenous fluids, thereby diluting the blood volume and reducing the likelihood of tumour cell detection. They showed the chance of intra-operative detection of tumour cells was halved by a blood loss of 0.5 l, and so excluded all patients with an intra-operative blood loss of more than 1 l from further statistical analysis. They found of the 7 patients who were positive pre- or post- but not intra-operatively, 5 (71%) had a blood loss of more than 1 litre, suggesting a possible dilution effect during the operation. In the articles reviewed, the degree of blood loss was only reported in the study by Weitz et al.

### B) What is the biological significance of CTC in CRC?

The detachment of malignant cells from the primary tumour is an early step in the formation of metastases [5]. The release of tumour cells is a continuous process [43], however the metastatic process is inefficient and the presence of tumour cells within the blood does not necessarily imply the subsequent development of metastases [44]. It is poorly understood which steps in the metastatic process are responsible for the inefficiency of tumour cells to form overt metastases.

Early studies suggested fewer than 0.1% of circulating tumour cells survive in the circulation [39], and only 0.01% form metastases [45]. It was thought the majority of CTC are removed from the circulation within 24 hours [39], by elimination in the first capillary bed they encounter [46]. However, recent work using cytoplasmically-labelled tumour cells (as opposed to nuclear-labelled cells which are more vulnerable to destruction) has found the majority of cells survive in the circulation for several days following injection, and the inefficient part of the metastatic process appears to be the variable growth of the cancer cell at the secondary site [47].

The metastatic process may be enhanced by the surgical procedure itself. It is known the entrapment of tumour cells in the microcirculation of a target organ is facilitated by the presence of fibrin and platelets [48,49]. The activation of coagulation that occurs during an operation may therefore augment this process [50]. Also, surgical stress has been shown to induce immune suppression [51], thereby increasing the metastatic efficiency.

The prognostic value of CTC in CRC has yet to be fully determined. Fujita et al [52] found patients negative preoperatively for CK-19 or CK-20 had significantly fewer recurrences and better 5-year disease free survival. Nakagoe et al [53] found a high sialyl Le (x) antigen or CEA in blood draining the tumour was an independent prognostic indicator of poor survival, and Yamaguchi et al [12] found a similar finding in patients positive for both CEA and CK-20. Other studies show conflicting results, however. Bessa et al [54] found a preoperative peripheral blood sample positive for CEA did not predict prognosis, and in a comprehensive review, Tsavellas et al [29] conclude, at present, the presence of CTC cannot be considered to be a reliable indicator of prognosis in any common solid malignancy because of the lack of large standardised trials with sufficient follow up.

### C) Is the no-touch isolation technique of CRC resection associated with an improved patient survival?

There are few studies directly comparing the outcomes following conventional and no-touch techniques. Turnbull et al [3] retrospectively compared 664 patients who underwent CRC resection using the no-touch technique against 232 patients with similar histological stage operated on by different surgeons employing the conventional method. They found the overall 5-year survival rate for the no-touch group was 51%, compared with 35% for the conventional group. They state the difference in mortality was due to an increased incidence of hepatic metastases in the conventional group. Subsequent criticism [4] of Turnbull's findings suggested patient selection may not have been random, and the basis of the no-touch technique incorporates an extended lymphadenectomy. The study also excluded cancers of the rectum.

Wiggers et al [4] randomly assigned 236 patients with tumours of the colon or rectum to a no-touch or conventional surgery group, and found no significant difference between the groups in terms of recurrence or survival. However, there was a trend towards fewer and later onset of liver recurrences in the no-touch group. Their recommendation was that the no-touch technique should be used for tumours where it is easily applicable.

The largest series was reported recently by Slanetz [55], who retrospectively reviewed 1863 cases of colorectal cancer resection over a period of 24 years. In 1050 cases, tumour mobilisation had occurred before regional mesenteric vessel ligation, whereas 813 cases had vessel ligation performed initially. The extent of mesenteric resection and tumour differentiation was reportedly similar between the two groups. The author reports the sequence of vessel ligation had little impact on the incidence of cancer-related deaths at 5 and 10 years, with no significant difference in survival rates between the early vessel ligation and conventional groups for colonic or rectal tumours. However, the sequence of vessel ligation did have a significant effect on the distribution of metastases. The early vessel ligation group was associated with fewer liver metastases but more systemic metastases compared with the conventional group. These findings may support the theory that after mesenteric pedicle clamping, there is a shift in draining blood into the systemic circulation [23]. There was also a significant increase in the local recurrence rate with the no-touch compared with the conventional technique (22.6% v 14.6%; p = 0.0001).

Another component of the no-touch isolation technique investigated by Slanetz is the control of intraluminal spread of malignant cells by applying bowel clamps or ligatures before tumour manipulation. He reviewed the results of 599 CRC resections in which bowel ligation prior to tumour mobilisation was used, and compared the data against 1416 resections in which bowel ligation was not performed. He found the application of bowel ligatures before tumour mobilisation significantly improved the 5-year cancer-related death rate for colon cancer (20% v 25%, p = 0.02), but not for rectal cancer. When considering colon and rectal cancer combined, bowel ligation prior to mobilisation significantly reduced the local (12% v 19%, p = 0.02) and distant (liver: 10% v 15%, systemic: 13% v 18%, p < 0.001) recurrence rates compared to resection without prior bowel ligation. These findings support the earlier work by Cole et al [56], who showed that ligatures around the bowel controlled the rate of local lymphatic and intraluminal dissemination of malignant cells.

## Conclusion

It is difficult to draw any firm conclusions from the articles studied due to the lack of standardisation of sample source, CTC detection method and the small sample sizes. However, the only study directly comparing conventional and no-touch surgical techniques found a conversion rate of 73% for conventional surgery and 14% for the no-touch technique, suggesting a benefit of vascular clamping before tumour mobilisation. The overall conversion rates for the other studies employing the no-touch technique were 0–16% (see Additional file 1), compared with 0–80% for studies utilising conventional surgery (see Additional file 2). Therefore, these data suggest there is a trend towards reduced tumour cell dissemination for the no-touch isolation method. The benefit of this in terms of improved patient survival, however, remains unproven. On purely theoretical grounds, the presence of alternate lymphovascular pathways for most colorectal tumours ensures complete isolation of the tumour-bearing segment is hard to achieve, limiting the technique's efficacy. In addition, the extent of mesenteric resection, and not the surgical technique employed, is probably the most important determinant of patient outcome following CRC surgery [55].

Due to the lack of consensus regarding the best technique for detection, the biological importance of CTC has not been fully determined. With the introduction of molecular biological techniques, the sensitivity for detection has improved considerably. The hope is that accurate detection of occult circulating malignant cells would help to stage the disease and predict prognosis, as well as monitor the response to therapy and highlight early the possibility of recurrence. However due to the differences in methodology and conclusions of studies investigating this area, the detection of CTC cannot, at present, be used to dictate therapeutic strategy.

Despite the methodological heterogeneity of the articles reviewed, we believe the introduction of highly sensitive methods of CTC detection has forced a re-analysis of the role of the no-touch isolation technique in CRC surgery, and the stages of CRC resection using the no-touch technique would appear to offer a sensible, systematic approach to the surgical management of large bowel tumours. However, further work needs to be done to investigate the remaining areas of uncertainty. In particular, future developments in CTC detection must ensure more automation and greater standardisation of techniques between centres. RT-PCR appears to offer the greatest sensitivity, and techniques detecting multiple markers that are more tumour-specific should be investigated [57,58]. The true biological importance of CTC in CRC needs to be assessed further, and finally, the actual benefit of the no-touch technique can only be determined by large scale randomised clinical trials utilising multiple venous sampling in patients matched for age and stage of disease.

## Competing interests

The author(s) declare that they have no competing interests.

## Authors' contributions

GA researched the topic and prepared the manuscript, IM conceived the article and AC critically analysed and formatted the manuscript.

## Supplementary Material

Additional File 1 Conversion rates for studies employing the no-touch isolation technique of CRC resection Click here for file

Additional File 2 Conversion rates for studies employing the conventional technique of CRC resection Click here for file
