# Supplementary material for: Colorectal cancer metastasis: in the surgeon's hands?
Source: Int Semin Surg Oncol. 2005 Feb 24;2:5. doi: 10.1186/1477-7800-2-5 (PMC553990; doi:10.1186/1477-7800-2-5)
Supplement: Additional File 1 — Conversion rates for studies employing the no-touch isolation technique of CRC resection [file 1477-7800-2-5-S1.doc]

**Abbreviations used for Additional Files 1 and 2**

**N** No-touch technique**CK-20** Cytokeratin 20

**C** Conventional technique**CEA** Carcinoembryonic antigen

**MASA** Mutant allele specific amplification**CGM2** Carcinoembryonic gene member 2

**NS** Not stated**GCC** Guanylyl cyclase C

**N/A** Not applicable**TC** Tumour cells

**PV**  Portal venous**WBCs** White blood cells

**SV** Systemic venous**LAC** Laparoscopically-assisted colectomy

**RT-PCR** Reverse transcription polymerase chain reaction**OC**  Open colectomy

**rt RT-PCR** Real time RT-PCR

**Additional File 1:** Conversion rates for studies employing the no-touch isolation technique of CRC resection

| **Guller**  **et al ^[16]6)^** | **Bessa**  **et al ^[17]7)^** | **Sales**  **et al ^[10]0)^** | **Weitz**  **et al ^[9]9)^** | **Hayashi**  **et al ^[8]8)^** | Reference |
| --- | --- | --- | --- | --- | --- |
| N | N | N | N | N  C | **Surgical technique** |
| 39 | 50  26 LAC  24 OC | 34 | 58 | 10  17 | **No of patients** |
| rt RT-PCR | RT-PCR | RT-PCR | RT-PCR | MASA | **CTC**  **detection method** |
| CEA or CK-20 | CEA | CEA and CGM2 | CK-20 | K-ras/p53 mutations | **Marker** |
| No | No | No | No | No | **Multiple samples?**  **(sample number )** |
| 10 TC/ml  blood | 1 TC/10^7^ WBCs | NS | 10 TC/10ml blood | ‘a few TC in thousands of WBCs’ | **Sensitivity** |
| 100% | 100% | NS | 100% | NS | **Specificity** |
| SV | SV  PV  (20 patients) | SV | SV | PV | **Sample**  **source** |
| 1/39 (3%) | OC SV 2/24 (8%)  PV 0/10 (0%) LAC SV 3/26 (12%)  PV 1/10 (10%) | 3/34 (9%) | *Overall*: 9/58 (16%)  *Blood loss < 1l*:  8/43 (19%) | 1/7 (14%)  8/11 (73%) | **Conversion rate**  **(all patients)** |
| 1/37 (3%) | OC SV 2/9 (22%)  PV 0/2 (0%)  *LAC*  SV 3/6 (50%)  PV 1/3 (33%) | 3/24 (13%) | *Overall*: 9/43 (21%)  *Blood loss < 1l*:  8/32 (25%) | 1/7 (14%)  8/11 (73%) | **Conversion rate**  **(patients negative preoperatively)** |
