# Supplementary material for: Colorectal cancer metastasis: in the surgeon's hands?
Source: Int Semin Surg Oncol. 2005 Feb 24;2:5. doi: 10.1186/1477-7800-2-5 (PMC553990; doi:10.1186/1477-7800-2-5)
Supplement: Additional File 2 — Conversion rates for studies employing the conventional technique of CRC resection [file 1477-7800-2-5-S2.doc]

**Abbreviations used for Additional Files 1 and 2**

**N** No-touch technique**CK-20** Cytokeratin 20

**C** Conventional technique**CEA** Carcinoembryonic antigen

**MASA** Mutant allele specific amplification**CGM2** Carcinoembryonic gene member 2

**NS** Not stated**GCC** Guanylyl cyclase C

**N/A** Not applicable**TC** Tumour cells

**PV**  Portal venous**WBCs** White blood cells

**SV** Systemic venous**LAC** Laparoscopically-assisted colectomy

**RT-PCR** Reverse transcription polymerase chain reaction**OC**  Open colectomy

**rt RT-PCR** Real time RT-PCR

**Additional File 2:** Conversion rates for studies employing the conventional technique of CRC resection

| **Ito**  **et al ^[15]5)^** | **Patel**  **et al ^[14]4)^** | **Tien**  **et al ^[13]3)^** | **Funaki et al**  **[20]0)** | **Yamaguchi**  **et al ^[12]2)^** | **Garcia-Olmo**  **et al ^[11]1)^** | **Mori**  **et al ^[19]9)^** | **Griffiths**  **et al ^[18]8)^** | Reference |
| --- | --- | --- | --- | --- | --- | --- | --- | --- |
| C | C | C | C | C | C | C | C | **Surgical technique** |
| 99 | 78 | 58 | 5 | 52 | 16 | 8 | ‘over 200’ | **No of patients** |
| rt RT-PCR | RT-PCR | RT-PCR | RT-PCR | RT-PCR | RT-PCR | RT-PCR | Cytology | **CTC**  **detection method** |
| CEA | CEA or  CK-20 | GCC | CK 20 | CEA and CK-20 | CEA | CEA | N/A | **Marker** |
| No | Yes (3) | Yes (2) | No | No | No | Yes (3) | Yes (2-6) | **Multiple samples?**  **(sample number)** |
| 10 TC/10^7^ WBCs | 1 TC/10^6^  WBCs | 10 TC/10ml blood | NS | 10 TC/2ml  blood | 10 TC/2ml  blood | 1-10 TC/10^7^ WBCs | NS | **Sensitivity** |
| 75% | 85-90% | 100% | 100% | 100% | 100% | 100% | NS | **Specificity** |
| SV | SV | SV  PV | SV | SV | SV | SV | SV | **Sample source** |
| Preop 38.4% +ve  Postop 50.5% +ve  (p=0.003) | 4/78 (5%) | 17/58 (29%)  14/58 (24%) | 4/5 (80%) | 5/52 (10%) | 0/16 (0%) | 1/8 (13%) | *Preop*: 2/50 (4%) +ve  *Intraop:* 50/100 (50%)+ve  *Postop*: 4/50 (8%) +ve | **Conversion rate**  **(all patients)** |
| N/A | 4/27 (15%) | 17/35 (49%)  14/27 (52%) | 4/4 (100%) | 5/49 (10%) | 0/14 (0%) | 1/6 (17%) | N/A | **Conversion rate**  **(patients negative preoperatively)** |
